# Temporal Changes in the Use of Wild Medicinal Plants in Trentino–South Tyrol, Northern Italy

**DOI:** 10.3390/plants12122372

**Published:** 2023-06-19

**Authors:** Giulia Mattalia, Felina Graetz, Matthes Harms, Anna Segor, Alessio Tomarelli, Victoria Kieser, Stefan Zerbe, Andrea Pieroni

**Affiliations:** 1 Institut de Ciència i Tecnología Ambientals (ICTA-UAB), Universitat Autònoma de Barcelona, 08193 Barcelona, Spain; 2 New York Botanical Garden, New York, NY 14058, USA; 3 University of Gastronomic Sciences, 12042 Pollenzo, Italy; felina.graetz@gmail.com (F.G.); matthes.harms@gmail.com (M.H.); segoranna@gmail.com (A.S.); alessio.tomarelli@gmail.com (A.T.); victoria.kieser@gmail.com (V.K.); 4 Faculty of Agricultural, Environmental and Food Sciences, Free University of Bozen-Bolzano, 39100 Bolzano-Bozen, Italy; stefan.zerbe@unibz.it; 5 Department of Medical Analysis, Faculty of Applied Science, Tishk International University, Erbil 44001, Iraq

**Keywords:** Alps, biocultural diversity, borders, ethnomedicine, historical ethnobotany, local ecological knowledge, mountain regions

## Abstract

Mountain regions are fragile ecosystems and often host remarkably rich biodiversity, and thus they are especially under threat from ongoing global changes. Located in the Eastern Alps, Trentino–South Tyrol is bioculturally diverse but an understudied region from an ethnobotanical perspective. We explored the ethnomedicinal knowledge of the area from a cross-cultural and diachronic perspective by conducting semi-structured interviews with 22 local inhabitants from Val di Sole (Trentino) and 30 from Überetsch–Unterland (South Tyrol). Additionally, we compared the results with ethnobotanical studies conducted in Trentino and South Tyrol over 25 years ago. The historical comparison revealed that about 75% of the plants currently in use were also used in the past in each study region. We argue that the adoption of “new” medicinal species could have occurred through printed and social media and other bibliographical sources but may also be due to limitations in conducting the comparison (i.e., different taxonomic levels and different methodologies). The inhabitants of Val di Sole and Überetsch–Unterland have shared most medicinal plants over the past few decades, yet the most used species diverge (perhaps due to differences in local landscapes), and in South Tyrol, people appear to use a higher number of medicinal plants, possibly because of the borderland nature of the area.

## 1. Introduction

Mountain regions often host rich biodiversity, and thus they are especially under threat from the ongoing global changes (e.g., [1]), particularly those related to climate and habitat shifts resulting from land-use changes during the last century [2]. European mountain ranges are no exception to such a trend (e.g., Gurung et al. [3] for the Carpathians, Miranda Cebrian et al. [2] for the Pyrenees, Rew et al. [4] for the Alps).

The Alps are one of the largest mountain ranges in Europe, and the Alpine region features complex historical, political, and ecological trajectories. For instance, the region of Trentino–South Tyrol in the Southern Eastern Alps was one of the last regions to be added to the country of Italy [5]. Until 1919, Trentino–South Tyrol was part of the Habsburg Empire, a political entity that was characterized by considerable linguistic and ethnic heterogeneity [6]. Despite attempts at “Italianization”, particularly under the Mussolini government (1922–1943), the region remained highly diverse in terms of culture and language. Accordingly, German, Italian, and Ladin coexist in this small mountainous territory [7,8]. In 1972, such diversity led to autonomy status for the provinces of Trento (mainly Italian-speaking) and Bozen–South Tyrol (mainly German-speaking).

Political and linguistic contexts often play a key role in the way people relate to plants (e.g., [9,10] for example from Bukovina, the easternmost region of the former Habsburg Empire). Visible (e.g., institutional/political) and invisible (e.g., cultural/linguistic) borders could offer valuable tools for detecting the effect of political, cultural, or linguistic heterogeneity on local ecological knowledge (LEK) (e.g., [9,11,12]).

The Alps have been often perceived as borders, but as well noted by Salsa [13], this mountain range has also served as zippers, as spaces for exchange among different socio-cultural groups. For instance, in the last ten years, five publications have addressed the ethnomedicine of cultural groups, three linguistic minorities and one religious minority of the Western Alps [14,15,16,17,18], and four addressed the folk medicinal uses of the Central Alps [19,20,21,22], and two of the Eastern Alps [23,24].

In addition to a spatial and socio-political perspective, a diachronic perspective could further contribute to our understanding of the complex dynamics of local ecological knowledge in the Alpine environment. Historical ethnobotanical studies in Europe have often focused on the eastern portion of the continent, where ethnobotanical archival data from the 19th and 20th centuries [25,26,27,28,29,30,31,32,33] are frequently available. These studies have generally shown a decrease in wild food plant-centered LEK, while local medicinal plant reports are not only fading but often re-arranged via influences of printed media.

In recent years, public interest in the use of traditional ethnobotanical knowledge and medicinal plants has steadily increased [34]. The cultivation and use of medicinal plants have become a growing market niche in the past few decades, which underlines the importance of folk medical knowledge for the promotion of ecotourism, eco-gastronomy, and organic farming [35]. This development could play an important role in conserving biodiversity and cultural heritage in South Tyrol and the Alps in general while revitalizing the relationship between humans and nature [36].

Despite the growing interest, the Trentino–South Tyrol region has been little studied from an ethnobotanical perspective compared to other Italian regions (see Cavalloro et al. [37] for an overview). Indeed, we found only four publications on the local ecological knowledge of medicinal plants in this region. Two studies listing the medicinal plants used by local populations were conducted over 25 years ago, in Val di Sole, Trentino [38], and the Puster Valley, South Tyrol [39]. Two more recent studies conducted, in several parishes of Trentino [37] in South Tyrol based on published material [36] have warned about the ongoing plant-related LEK erosion. Additionally, the concept of Cultural Keystone Species with regard to traditionally used medicinal plants was studied by Petelka et al. [40] and interviews were conducted in South Tyrol by Scherrer et al. [41] to gain insight into, and reflections on, the cultural value of traditional medicinal plants and their interplay within the local landscape, nature conservation and their role in environmental education and knowledge transfer across generations.

Although under-researched, Trentino–South Tyrol is rich in cultural, linguistic, and ecological diversity. Additionally, considering the peculiar vulnerability of the Alpine context and the current threats from climate change and social changes, documenting the LEK held by culturally and linguistically distinct groups could be crucial to fostering future biocultural conservation strategies. Therefore, the objective of this study is to explore LEK related to medicinal plants from a cross-cultural and diachronic perspective and, specifically:

1. to document current LEK related to wild medicinal plants and its transmission strategy in Val di Sole (Trentino) and South Tyrol,
2. to identify similarities and differences with historical ethnomedicinal data regarding Trentino and South Tyrol published over 25 years ago, and
3. to identify similarities and differences among current LEK held by inhabitants of Val di Sole and South Tyrol.

## 2. Results

### 2.1. Current Use of Wild Medicinal Plants in Überetsch–Unterland (South Tyrol)

We documented the use of 73 plant species, belonging to 30 families (including 29 gymnosperms and an angiosperm), and one lichen (Table 1). The dominant plant families were Asteraceae with 13 species, followed by Lamiaceae with 11 species, and Rosaceae with eight species. Among the mentioned medicinal plants, six were cultivated (*Ocimum basilicum*, *Salvia rosmarinus*, *Tropaeolum minus*, *Mentha* × *piperita*, *Levisticum officinale*, *Melissa officinalis*) while eight were partially cultivated (*Calendula officinalis*, *Lavandula angustifolia*, *Salvia officinalis*, *Borago officinalis*, *Alchemilla vulgaris*, *Ribes nigrum*, *Thymus vulgaris, Phyllanthus niruri*); all other plants were collected in the wild.

Interviewees reported that medicinal plants can cure numerous illnesses.

Different parts of medicinal plants, especially the aerial parts, leaves, and flowers, were used for the preparation of a variety of remedies.

The interviewees indicated that the collected medicinal plants are mainly used to prepare tinctures, oil, infusions, salves, teas, and powders. Herbs are also sometimes simply mixed and used in smoothies or juices (i.e., *Urtica dioica* and *Plantago lanceolata*). Jams and syrups are made from a variety of fruit, berries, and herbs. Examples include jam made from *Sambucus nigra* berries, which have antiviral and antimicrobial effects, and jam made from *Rosa canina* fruit to supplement vitamin C. Syrups are made, for example, from *Salvia rosmarinus* and *Lavandula angustifolia*. Many respondents replace table salt with aromatic herb-based salts as a healthier seasoning. Additionally, bath salts or bath additives are prepared with herbs such as *Thymus vulgaris*, *Plantago major*, *Urtica dioica,* and *Valeriana officinalis*.

The three medicinal plants most frequently mentioned by our respondents were *Urtica dioica* (13 interviewees), *Achillea millefolium* (12), and *Calendula officinalis* (10). The most commonly mentioned plant, *Urtica dioica*, is considered a good source of iron for the body. It detoxifies, refreshes the brain, strengthens the immune system, is a diuretic, induces labor during pregnancy, and supports women with menstrual and hormonal disorders. The plant can be dried and used as a tea or bath additive, freshly squeezed or blended as a juice or smoothie, and used, as is common, as a substitute for spinach in traditional dishes such as “Spätzle” (special kind of egg noodle) or “Knödel” (dumplings). Participants also mentioned some knowledge about nettle, possibly arising from popular media, i.e., that the root, which can be eaten dried or fresh, has good properties for the liver, and that the seeds are rich in nutraceuticals. *Achillea millefolium* was described by the interviewees as having various positive effects on the body. It not only has a calming and antidepressant effect but also relieves stomach pain, detoxifies the body, stimulates the appetite, helps women with menstruation and menopause, and contains all the necessary trace elements. According to our interviewees, the whole plant can be cut approximately a hand’s width from the ground during the flowering period, tied into a bouquet, and dried upside down. *Achillea millefolium* can also be used as a tea, as a bath infusion for menstrual cramps, as a culinary mineral salt, for example in soups, and as a seasoning powder. The third most frequently mentioned plant was *Calendula officinalis*, which is said to be good for digestion and to help with diarrhea. It can be applied to closed wounds to promote healing and improve scarring. *Calendula officinalis* also helps to refresh the skin. As another external treatment, it can be applied as a cream or oil on bruises. The herbs are infused in oil for at least three weeks, which can then be further processed into a cream. The infused oil can also be consumed to alleviate stomach pain. Finally, it is often prepared as a tea.

Our interviewees gave several pieces of advice on the proper use of medicinal herbs. For instance, several interviewees advised that, when infusing medicinal herbs, it is important to steep them only briefly, usually about two to three minutes, so as not to extract the wrong active ingredients and burn the herbs. Infused oils and creams should not be stored for too long to avoid rancidity. It was also recommended that attention be paid to the correct dosage of the active ingredients, as interviewees know that overdoses may have harmful or undesirable side effects.

In South Tyrol, several interviewees stated that gathering medicinal herbs was part of their childhood, a family activity associated with hiking in the region’s mountains. Most often, mothers and grandmothers possessed the traditional knowledge of local medicinal plants. Many participants mentioned a well-known saying of their grandmothers: “For every ailment, there is an herb”. From the recollections of our interviewees, we learned that many of these collected plants were subsequently incorporated into a variety of dishes cooked by their mother or grandmother and that they were also used to treat numerous diseases. Thus, female family members were often the primary source of their knowledge and interest in traditional medicinal herbs. Knowledge of local medicinal herbs was acquired not only from female family members, but also through a long-standing personal interest in wild plants that led to self-study via books, literature, or participation in formal courses, lectures, workshops, and seminars. The young people we interviewed liked to use books to acquire knowledge about medicinal plants, as did elderly individuals who used many different “Kräuterbüchlen” (literally: books about herbs). When reviewing the utilized literature, it is evident that it consisted mostly of writings by the authors Maria Treben, Hildegard von Bingen, and Gottfried Hochgruber. These authors enjoy a great reputation, mainly because of the success respondents have experienced healing themselves using their books.

### 2.2. Current Use of Wild Medicinal Plants in Val di Sole, Trentino

We documented the use of 36 species belonging to 21 families (20 angiosperms and a gymnosperm). The dominant families were Asteraceae with seven species, followed by Pinaceae with five species, and Rosaceae with four species. Among the mentioned medicinal plants, one was cultivated (*Laurus nobilis*) and three were semi-cultivated (*Alchemilla* spp., *Corylus avellana*, *Ficus carica*).

According to our interviewees, cough and other respiratory ailments were the most commonly cured with medicinal plants, along with problems of the digestive and integumentary systems. The top-mentioned medicinal plants included *Larix decidua* (12 interviewees) and *Malva sylvestris* (11), followed by *Sambucus nigra*, *Humulus lupulus*, *Gentiana lutea*, and *Pinus mugo* (8). The recipes of the mentioned preparations do not include exact measurements, as the preparations are never written down but rather passed on orally through generations; the required amount is often simply “a handful”, “a bit”, or “a basket”.

One example of current medicinal use is the collection of the green buds of mountain pine (*Pinus mugo*) for the preparation of a cough syrup to be used during the winter months. Additionally, Icelandic moss (*Cetraria islandica*), a species that grows close to the ground in only a few high-altitude areas, is used to alleviate coughing and bronchitis when boiled with honey-sweetened milk. The most used and important root is that of gentian (*Gentiana lutea*). Interviewees reported the use of this root to flavor digestive liqueurs and aquavits. After the collection of the root between September and October, the cut-up roots are put into white aquavit for about three weeks. The end product is still frequently consumed and loved by the valley population. The root was once also used to prepare a depurative decoction to be drunk, especially by women, during the seasonal change from winter to spring.

Another fundamental ingredient was extracted from the responses of the interviewees: larch resin, “l’Argà” or “Resina del Lares” in the local dialect. This ingredient, mainly used in creams and unguents, is collected by a single man in the entire valley, and according to him, he is the last man in the world known to do this job. Larch resin collector, or “Largaiòl”, is a laborious profession: each year larch trees are hand drilled at the base; the hole, around 40 cm in length, is then closed with a larch wood cork. After three to four years, the “Largaiòl” will then collect the resin with a special tool called a “sgorbia” and proceed to filter the resin. One larch tree produces, on average, 100 g of resin every four years, making the job extremely strenuous and time-consuming. The most widespread preparation with larch resin is “l’Ont dei Tai”, an unguent used to remove splinters or to help the healing of infected cuts. Larch is also used to help alleviate coughs and colds by warming, in a double boiler, the resin and breathing in the steam while covering the head with a cloth.

Another well-known and used plant is dandelion (*Taraxacum* sect. *Taraxacum*). Depending on the area, the dialectal name changes from “zicoria” or “cicoria” to “dente de cagn” or “dente di leone”. Young dandelion stems, which are less bitter, are eaten raw in salads. More mature and larger leaves are used to prepare a blood and liver depurative decoction. This plant was once picked everywhere, even along roadsides and within villages; however, today, because of pollution and agricultural expansion, it must be picked from other locations, far from roads and settlements to avoid pesticides and smog pollution. In the kitchen, dandelion is used to prepare gnocchi, frittata, and canederli (a regional dish of boiled dumplings). The consumption of this plant is so rooted in valley tradition that every year in April there is a festival called “Zicoria in Val di Rabbi”.

In traditional phytotherapy, buds and young pinecones of mountain pine, larch, and stone pine, believed to have the highest concentration of medicinal properties, are used to prepare bud extracts. Bud extract is a product obtained through a 15- to 20-day maceration in alcohol and glycerine. After the maceration period, the liquid is filtered and bottled. The bud extracts are mainly used to strengthen immunity or as depuratives. As with all phytotherapy practices, the bud extracts need prolonged consumption, even two to three months, to have their full effect. Bud extracts are to be taken up to three times per day in variable dosages, in drops (from four to ten) under the tongue. Another method of extraction is tincture, in which the fresh plant is macerated in a solution of water and alcohol for up to 30 days. After this period, the liquid is then filtered and bottled. A more homemade and easier preparation is sugar syrups: pinecones and buds are put in jars with sugar and exposed to sunlight for 30 days to obtain a thick and aromatic syrup used to treat coughs and sore throats.

Even though the number and variety of plants and their curative properties are numerous, most of the interviewees use phytotherapy preparations in conjunction with conventional medicine. For smaller, less severe problems they seem to prefer phytotherapeutic methods, often reducing the severity of the illness via the consumption of a tincture, decoction, or bud extract. Most of the interviewees only gather plants near their houses, while a few individuals travel to specific areas and locations to pick only the best quality ingredients for traditional preparations.

In Val di Sole, the interviewees showed a strong interest in the gathering and usage of wild plants. Much of the information was learned from their parents and grandparents. Some of them reported gaining a renewed interest in wild plants following classes held by Eulalia Panizza, a local naturopath and important teacher in the valley.

### 2.3. Diachronic Comparison with Historical Sources

**Figure 1:**
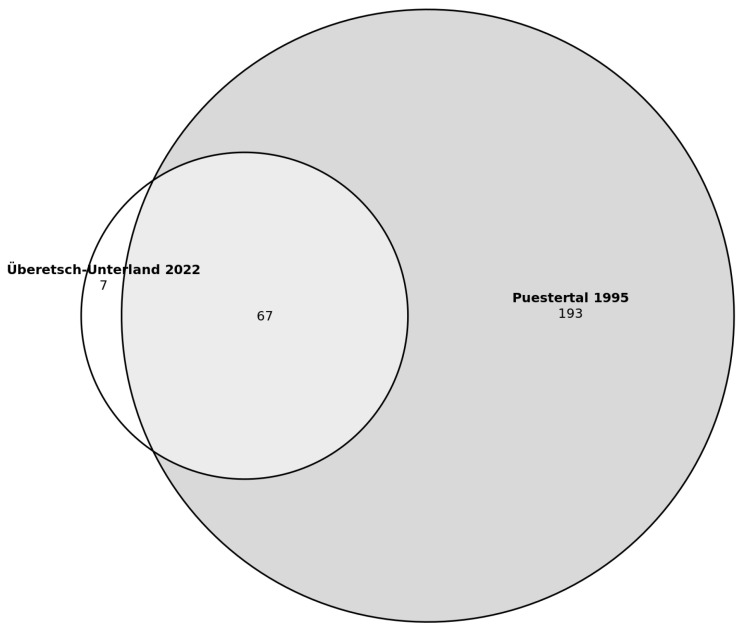
Venn diagram of the number of medicinal plant species in the Puster Valley (presumably collected in 1994) and Überetsch–Unterland (collected in 2022).

The comparison between medicinal plants mentioned in South Tyrol in summer 2022 (in Überetsch–Unterland) and those reported by Pick-Herk in 1995 [39] (in the Puster Valley) revealed that 67 species (out of a total of 74, corresponding to about 90%) were also reported in the past for medicinal use (Figure 1). Thus, only seven plants (*Leontopodium nivale*, *Dipsacus fullonum*, *Lamium galeobdolon*, *Tropaeolum minus*, *Leonurus cardiaca*, *Centaurea cyanus*, *Borago officinalis*) were not reported by Pick-Herk [39]. The Jaccard Index is 25.

**Figure 2:**
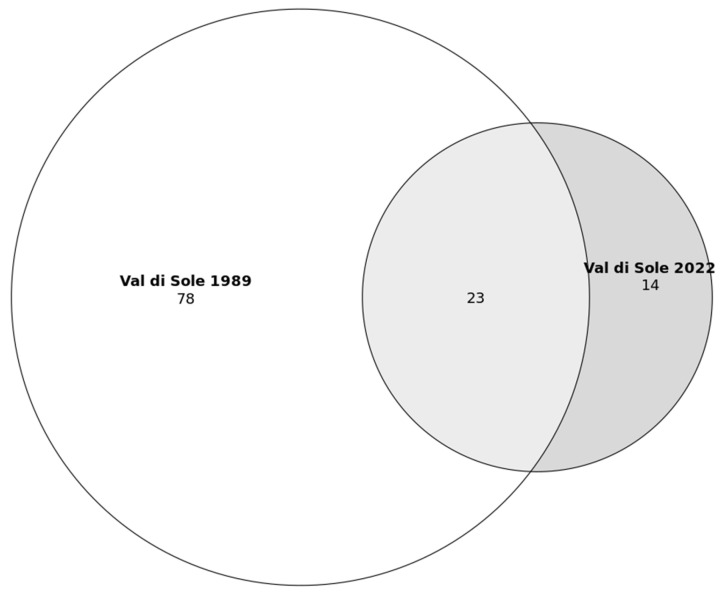
Venn diagram of the number of medicinal plant species in Val di Sole (collected in 1978–1979 and 2022).

The comparison between plants mentioned in the study of Cappelletti and Fanzago [38] and our research in Val di Sole revealed that 23 species (out of a total of 37, corresponding to about 60%) were also reported in the past (Figure 2). Thus, 14 species currently used were not reported as used in the past in Val di Sole. The most common species mentioned now but not in the past are *Alchemilla alpina*, *Plantago lanceolata*, *Crataegus monogyna*, *Tilia platyphyllos*, *Ficus carica*, *Phyllanthus niruri*, *Symphytum officinale*, *Pinus cembra*, *Rhodiola rosea*, *Artemisia umbelliformis* subsp. *umbelliformis*, *Malva sylvestris*, *Salix* spp., *Gentiana lutea* and *Tilia cordata*. The Jaccard Index is 20.

### 2.4. Cross-Cultural Comparison

**Figure 3:**
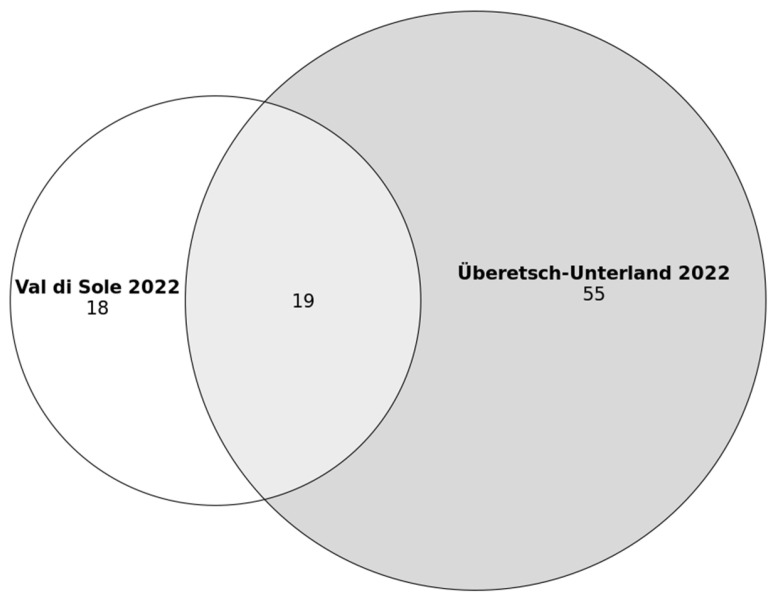
Venn diagram of the number of medicinal plant species in Val di Sole and Überetsch–Unterland (collected in 2022).

**Figure 4:**
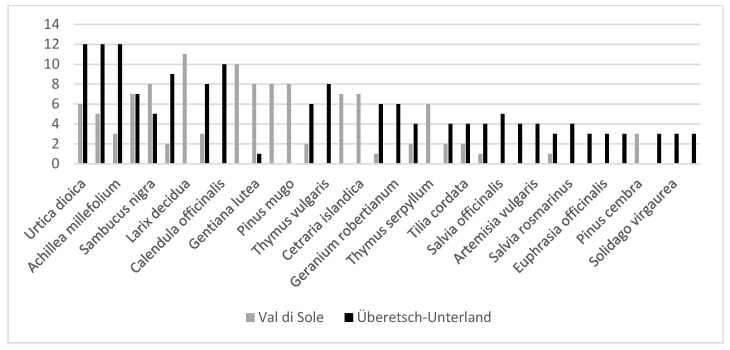
Comparison of plant taxa used by at least three interviewees in Val di Sole and Überetsch–Unterland.

Nineteen medicinal plants were mentioned in both Val di Sole (Trentino) and Überetsch–Unterland (South Tyrol) (Figure 3). Twenty-one percent (*n* = 19) of the mentioned plant species were currently common to the two groups, while 20% (*n* = 18) were mentioned only in Val di Sole and 59% (*n* = 55) only in Überetsch–Unterland. The Jaccard Index is 21. The most common taxa include *Urtica dioica*, *Taraxacum* sect. *Taraxacum*, *Achillea millefolium*, *Hypericum perforatum* and *Sambucus nigra*. However, none of the top three mentioned plants are common to the two communities (Figure 4). Indeed, the top-cited species in Val di Sole was *Larix decidua* (11 interviewees), followed by *Malva sylvestris* (10) and *Pinus mugo*, *Humulus lupulus* L., *Sambucus nigra*, and *Gentiana lutea* (8 each). In Überetsch–Unterland, *Urtica dioica*, *Taraxacum sect. Taraxacum* and *Achillea millefolium*. were the most used plants (12 interviewees mentioned each plant), followed by *Calendula officinalis* (10) and *Arnica montana* (9).

**Figure 5:**
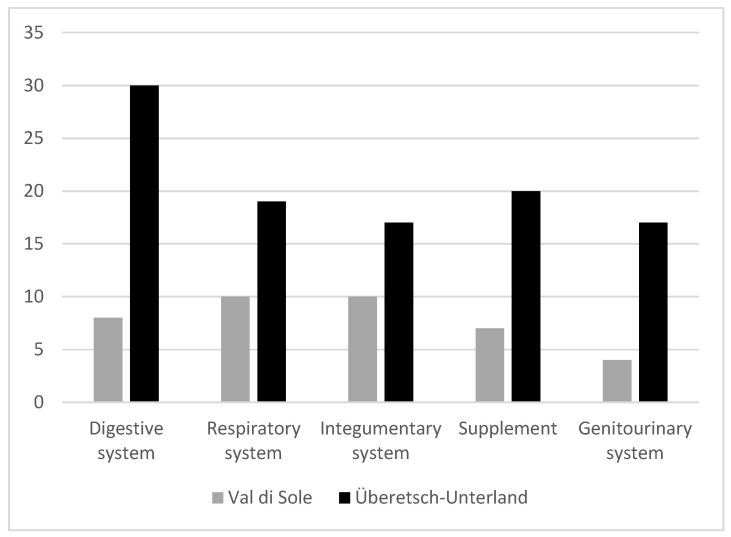
Top five apparatuses per number of mentioned ailments. Supplement refers to substances used as antiviral, antibacterial, vitamin and mineral supplements, energizers, etc. which do not refer to any specific apparatus.

The most frequently reported medicinal uses were for the digestive system (e.g., healing stomach and intestine) and the respiratory system (bronchitis, sore throat, cough, cold) (Figure 5). Moreover, supplements play an important role and several plants are used to this scope. In both Val di Sole and South Tyrol medicinal plants are also used to treat integumentary and genitourinary systems (although this was less relevant in Überetsch–Unterland).

**Figure 6:**
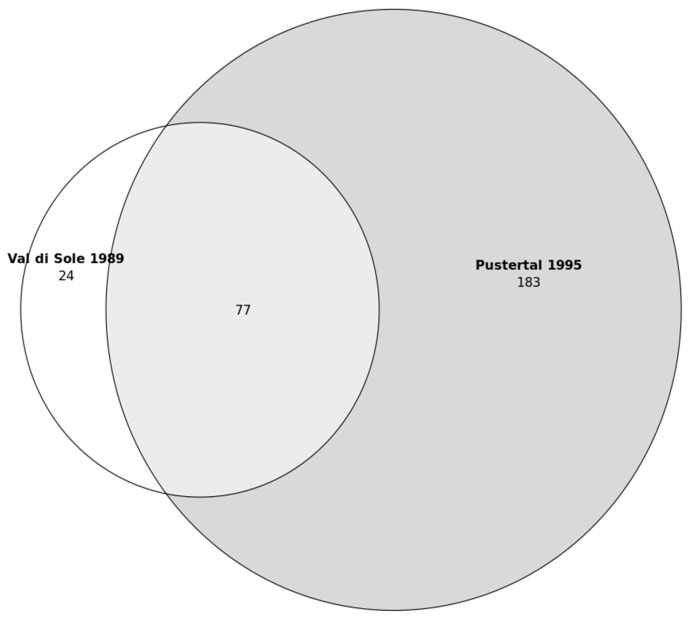
Venn diagram of the number of medicinal plant species in Val di Sole (collected in 1978–1979) and the Puster Valley (presumably collected in 1994).

The cross-cultural comparison of the historical sources reveals that 27% (*n* = 77) of the mentioned plant species were common to the two groups, while 8% (*n* = 24) were mentioned only in Val di Sole and 65% (*n* = 55) only in the Puster Valley (Figure 6). The Jaccard Index is 27.

## 3. Materials and Methods

### 3.1. Study Area

**Figure 7:**
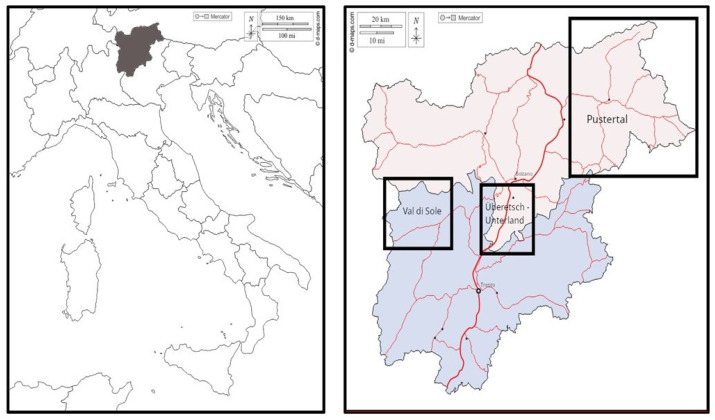
On the left, the Autonomous Provinces of Trento and Bozen–South Tyrol in northeastern Italy and, on the right, the location of the three case study areas of Val di Sole, Überetsch–Unterland, and Puster Valley (the latter for the historical comparison).

**Figure 8:**
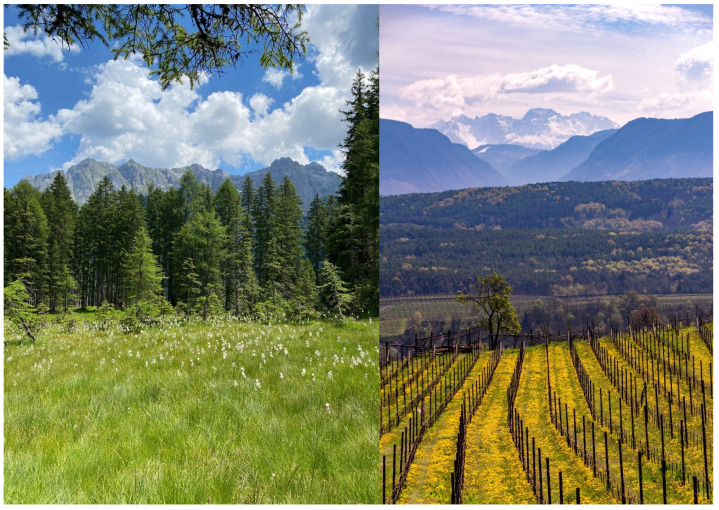
On the left, a typical panorama of Val di Sole (credit: Anna Segor); on the right, a typical view of Überetsch–Unterland (credit: Lena Seebacher).

Val di Sole in the Autonomous Province of Trento is the largest branch of Val di Non in northwest Trentino (Figure 7 and Figure 8). The valley floor extends from 700 m a.s.l. in Malè to over 3500 m a.s.l. on the mountain peaks that surround the valley. The population of Val di Sole, called Solander in the local dialect, totals approx. 15,000 inhabitants. Each of the 13 municipalities is characterized by numerous hamlets that were historically all independent municipalities. Even if the size of the population has not changed much, Val di Sole has changed drastically since the end of the Second World War and the economic boom that followed. Land-use changes, including the expansion of cultivated areas and the intensification of agriculture (particularly for apple, wine grape, and legume production), the opening of large ski resorts and the construction of new infrastructure (e.g., roads, hotels, mountain bike paths), have shaped the valley considerably.

Überetsch–Unterland (*Oltradige-Bassa Atesina* in Italian) is a district in the southern part of the Autonomous Province of Bozen–South Tyrol (Figure 7 and Figure 8). It consists of 18 small municipalities (ranging from 393 to 18,000 inhabitants each). Among the 76,000 total inhabitants, about 67% belong to the German language group and 32% to the Italian language group [42]. The area is well known for its intense viticulture and fruticulture. With almost two million overnight stays per year, tourism makes an important contribution to income generation [42].

The main characteristics of the two studied valleys are summarized in Table 2.

### 3.2. Data Collection

Primary data were collected in July 2022. Interviews were carried out in both “Bezirksgemeinschaft Überetsch–Unterland” (“Comunità Comprensioriale Oltradige-Bassa Atesina”) in South Tyrol, consisting of 18 municipalities, as well as Val di Sole, and more specifically the villages of Folgarida, Malga di Dimaro, Dimaro, Mastellina, Costa Rotian, Castello, Pejo, Malè, Presson, Monclassico, San Bernardo in Val di Rabbi, and Pracorno. Ethnobotanical information was collected through qualitative semi-structured interviews with a total of 52 interviewees (30 in Bezirksgemeinschaft Überetsch–Unterland and 22 in Val di Sole) chosen via convenient sampling. The main recruitment criterion was expertise in local medicinal plants. No special consideration was given to socio-demographics, such as age, income, or education level. Personal contacts were used to select the first individuals for interviews, and then we used the snowball method [43]. We interviewed the local population using ten open-ended questions about their current and past use of medicinal plants by mentioning one part of the body at a time. We strictly followed the ethical guidelines of the International Society of Ethnobiology [44]. Prior and Free Informed Consent was obtained before starting the interviews, which were conducted in German in South Tyrol and Italian in Val di Sole, with respondents using dialect and vernacular names. Verbal informed consent was always obtained before each interview, with the purpose of the study clearly stated; interviewees’ data were later anonymized. The interviews were mostly conducted in person, although the location varied according to the respondents’ wishes. In some cases, when informants invited us to their homes, they showed us dried parts of medicinal plants or gave us “Schnaps” (liqueur) or tinctures to taste. Because of the COVID-19 pandemic which started in 2020, some interviews had to be conducted by telephone. Depending on the expertise of the interviewees, the interviews lasted between 15 and 60 min. Upon agreement, the interviews were recorded. Botanical identification was carried out linking the local plant names to those recorded in previous field ethnobotanical studies (see following paragraph) that were conducted in the same area a few decades ago and in which proper taxonomic identifications were performed [38,39]. For the very few new taxa quoted in the current study only the identification was presumed upon their quoted common Italian and German plant names.

Secondary data for conducting the diachronic analysis were collected through a literature search of studies conducted through semi-structured interviews over 25 years ago in the area. This resulted in the discovery of an article pertaining to the plant-based ethnomedicine of Val di Sole, published in Italian in 1989 [38]. Unfortunately, no ethnobotanical data were available for the Überetsch–Unterland area, so we used the closest historical ethnobotanical research, which corresponded to a master thesis about medicinal plants of the Puster Valley completed at the University of Wien in 1995 [39]. The Puster Valley is in the northeastern region of South Tyrol (Figure 1), and it is a German-speaking valley, with a minority of Ladin-speaking people. Further details of the methodology of these sources are available in Table 3.

### 3.3. Data Analysis

After conducting the interviews, we organized the data into two Excel tables, one for each of our case studies. For each plant taxon, we provided the vernacular names, the botanical family to which it belongs, how often our respondents mentioned it, whether it is a wild or cultivated medicinal plant, where it is most commonly found, what parts are used, and what medicinal uses it has. Plant species and families were verified against Plants of the Word Online and APG IV [45,46]. Medicinal uses were checked against the categories of ICD-11 [47] whenever possible. The tables were then merged, and two more columns were added to compare the obtained data with the historical data. In this case, we considered that use was also reported in historical data if the same species was reported with a medicinal purpose. We then used an online freely available tool for drawing Venn diagrams [48] and we calculated the Jaccard Index according to González-Tejero et al. [49].

## 4. Discussion

The results reveal two main findings. First, 75% of the plants currently in use were also used in the past. Second, the inhabitants of Val di Sole and Überetsch–Unterland have shared most of the local ecological knowledge related to medicinal plants over the past few decades, yet the most used species diverge, and in South Tyrol people appear to use a higher number of medicinal plants.

Before discussing our results, we would like to critically elaborate on the diachronic methodology. Although temporal comparison with previous studies is a valuable tool for better understanding the evolution of LEK, the methodology used (especially sample selection, the number of interviewees, and methods of data collection) is often vaguely described or altogether absent, which hampers precise comparability of the studies. For instance, in our comparison, we are missing information regarding the number of informants for the research conducted in Val di Sole in 1978–1979 [38] and the year of data collection for Pick-Herk [39]. Moreover, even when mentioned, data collection is rarely precisely described, thus undermining replicability. For instance, Pick-Herk [39] reported obtaining data from expert individuals, including farmers, monks, and midwives, without clearly describing their percentages and whether they reported knowledge acquired in the area. Finally, the evolving socio-economic context presents the interviewees and researchers with a different relationship given the different access to information and especially social media now and in the past.

The first finding relates to diachronic continuity in the use of medicinal plants in the studied areas, especially as most of the medicinal species reported by our interviewees in the summer of 2022 were reported in previous studies. This is not surprising considering that knowledge is mainly vertically transmitted. In South Tyrol, only 9% of the mentioned species were not reported in the past, while in Trentino 38% were not reported. We argue that various factors could have led to the adoption of “new” medicinal species. First, they could have been introduced through social media and other bibliographical sources; this could be the case for the use of *Tropaeolum minus*, *Leonurus cardiaca*, *Centaurea cyanus*, and *Borago officinalis*. In Trentino, the use of medicinal plants could have been influenced by the long-term activity of local herbalist Eulalia Panizza and her writings (which could be the case for *Alchemilla alpina*, *Phyllanthus niruri*, *Symphytum officinale*, *Rhodiola rosea*, and *Gentiana lutea*). Second, different taxonomic levels or species belonging to the same genus were reported for the same ailments. For example, in South Tyrol, *Leontopodium nivale* (Ten.) A. Huet ex Hand.-Mazz. was mentioned in our research, while *Leontopodium nivale* subsp. *Alpinum* (Cass.) Greuter was reported in historical sources. In Trentino, *Crataegus monogyna* Jacq. was not included in historical sources where only *Crataegus laevigata* (Poir.) DC. was mentioned. Similarly, *Tilia platyphyllos* Scop. and *Tilia cordata* Mill. were observed in 2022, while *Tilia* × *europaea* L. was reported by Cappelletti and Fanzago [38]. Third, the discrepancy between past and current medicinal species could be due to the methodology applied in the studies (our data collection in Val di Sole did not include cultivated plants, while Cappelletti and Fanzago [38] did include them). Finally, some species which are very important now, such as *Pinus cembra* L. in Trentino, were not mentioned in the past, possibly because of the recent expansion of forests as a consequence of the abandonment of mountain grasslands.

The second result indicates a similarity between the two groups as they show a fair amount of overlapping of medicinal plant use (19 taxa, corresponding to a fifth of the total). These include easily recognizable species with wide distribution, availability, and versatility (e.g., *Urtica dioica*, *Taraxacum* spp., *Hypericum* spp., *Equisetum arvense*, *Matricaria chamomilla*, *Rosa canina*) and other species specific to the Alpine region (*Arnica montana*, *Alchemilla vulgaris*, *Gentiana acaulis*). Despite some species being listed in the South Tyrolean Red Book [50], these seem to be popular medicinal plants in different areas of the Alps (e.g., [15,20,23,51]). The findings also reveal divergences in the most used plants. This may be due to differences in the landscape, as Val di Sole is mainly forested and thus conifers such as *Larix decidua* and *Pinus mugo* play an important role. These taxa, however, were not among the species mentioned in Überetsch–Unterland, likely because most of the interviewees live in lower altitude areas where agricultural land use dominates. Overall, our study found a richer corpus of local ecological knowledge in South Tyrol, which is consistent with the comparison of historical sources. This may be due to the intrinsic cultural nature of the area lying between the Italian and Mediterranean sphere and the German and Continental sphere from a geographical, linguistic, and cultural perspective. Such blending may have led to a richer corpus of knowledge that draws from these cultural, historical, geographical, and linguistic areas.

Our results are only partially in line with the findings of two recent studies conducted in the two autonomous provinces. In 2020 and 2021, Cavalloro et al. [37] interviewed 200 people across Trentino about their herbal ethnomedicinal knowledge. Two of the top five used plants mentioned in that study [37] (*Pinus mugo* and *Malva sylvestris*) were also reported by our participants in Val di Sole, while the other mentioned species were common ethnomedicinal remedies (*Achillea millefolium*, *Arnica montana*, *Hypericum perforatum*, *Larix decidua*, *Sambucus nigra*, *Gentiana lutea*). One top species in each study group was not mentioned in the other (*Satureja montana* was not mentioned in Val di Sole, while *Humulus lupulus* was mentioned in Val di Sole but not reported in the study by Cavalloro et al.).

Petelka et al. [36] conducted a literature review of the medicinal plants used in South Tyrol. The number of medicinal plants reported by Petelka and colleagues is much larger than the number of ethnomedicinal plants we found in Überetsch–Unterland (255 versus 74), possibly due to differences in methodology. We refrain from quantitative analysis because it is particularly difficult to compare data obtained from regional reviews with those obtained through semi-structured interviews conducted in more restricted areas. Among the most frequently cited species, one—*Urtica dioica*—was also among the most used species in the review by Petelka et al. [36], while the others were mentioned in both studies but were not among the most cited species in the other study (*Hypericum perforatum*, *Plantago lanceolata*, *Taraxacum* sect. *Taraxacum* and *Achillea millefolium*).

The study has, however, two main limitations: as in the majority of historical ethnobotanical investigations, the set of interviews and the area in which we conducted them was not large, and the actual adopted field methods may have not been the same as those in the historical research, as the earlier works used in the comparative analysis were published at a time when it was not yet common to precisely describe how the study participants were recruited (sampling) or how the interviews were conducted. This study sheds light on the importance of local ecological knowledge as an intangible heritage which nevertheless is undergoing a process of hybridization with different written sources of knowledge including social media and books published in Italian and/or German.

## 5. Conclusions

This diachronic and cross-cultural comparison of the ethnomedicine of Trentino and South Tyrol reveals that about 75% of the plants currently in use were also used in the past and that the inhabitants of Val di Sole and Überetsch–Unterland have shared the majority of medicinal plants over the past few decades, yet the most used species diverge and, in South Tyrol, people appear to use a higher number of medicinal plants. This highlights the dappled pattern of local medicinal plant use in a small but culturally diverse Alpine region. Indeed, mountain regions not only host rich biodiversity but are also often reservoirs of local ecological knowledge, which could serve as a basis for the sustainable use of local natural resources and local small-scale circular economies. For instance, in Trentino and South Tyrol, some smallholders run local businesses cultivating and foraging medicinal plants (e.g., Bergila in the Tures Valley or Schmiedthof in the Isarco Valley), or such local ecological knowledge is taught in seminars and activities for children and adults (e.g., the mountain school of Eulalia Panizza in Vermiglio, Val di Sole). Moreover, the revitalization of local wild plant-centered LEK should be further fostered by similar bottom-up initiatives.

Considering the complex biological and cultural diversity of this mountain area, more research is needed to further and better document the diachronic evolution of local medicinal plant knowledge as this could be pivotal in valorizing (and preserving) its embedded biocultural diversity.

## Figures and Tables

**Table 1:** List of the quoted botanical taxa, their folk names, used parts, the local preparations, and medicinal uses/treated illnesses (as reported by the emic perceptions that study participants mentioned), and the frequencies of quotation in the two study areas. [C: cultivated; W: wild; * also in the corresponding historical source].

Botanical Taxon and Family	Local Vernacular Names	Status	Part(s) Used	Trentino Frequency of Quotation	Südtirol Frequency of Quotation	Preparation	Local Perceived Medicinal Uses or Treated Illnesses
*Abies alba* Mill. (Pinaceae)	Abete bianco, Avez (T)	W	Buds	2 *		Bath, tincture	Chilblains, bones, and tooth support
*Achillea erba-rotta* subsp. *moschata* (Wulfen) Vacc. (Asteraceae)	Medico gentile, Erba iva (T)	W	Flowers	7 *		Schnaps, tea	Digestive, cramps
*Achillea millefolium* L. (Asteraceae)	Achillea millefoglie, Milifoi (T), Schafgarbe, Jungfrauenkraut, Garbenkraut (S)	W	Flowers	3 *	12 *	Oil infusion, tea, fresh juice, decoction	Cicatrizing, disinfectant, soothing, digestive, depurative, menstrual pain, power source, mineral salt source, antibacterial, antibiotic, anti-inflammatory, arthritis, fever, gastrointestinal cramps, wounds
*Acorus calamus* L. (Acoraceae)	Kalmus (S)	W	Roots		1 *	Tea, tincture, oil	Digestive disorders, respiratory diseases
*Aegopodium podagraria* L. (Apiaceae)	Gewöhnlicher Geißfuß, Giersch (S)	W	Aerial parts of the flowering plant		4 *	Tea	Rheumatism, gout
*Agrimonia eupatoria* L. (Rosaceae)	Odermennig (S)	W	Aerial parts of the flowering plant		1 *	Tea, tincture	Inflammation in the oral cavity, diarrhea
*Alchemilla vulgaris* L. (Rosaceae)	Frauenmantel (S)	W, C	Aerial parts		2 *	Tea	Menstrual and hormonal disorders
*Alchemilla xanthoclora* Rothm. (Rosaceae)	Alchemilla (T)	W, C	Aerial parts	2 *		Tea	Stomach cramps
*Allium ursinum* L. (Amaryllidaceae)	Bärlauch (S)	W	Flowers, leaves, bulbs		2 *	Tea, pesto, salt	Arteriosclerosis, hypertension, digestive disorders
*Althaea officinalis* L. (Malvaceae)	Eibisch, Weiswurzel (S)	W	Flowers, leaves, roots		1 *	Tea, tincture, syrup	Bronchitis, stomach, and intestinal inflammation
*Angelica archangelica* L. (Apiaceae)	Engelwurz (S)	W	Roots, leaves		1 *	Tea	Digestive disorders
*Angelica sylvestris* L. (Apiaceae)	Engelwurz (S)	W	Roots, leaves		1 *	Tea	Digestive disorders
*Armoracia rusticana* G.Gaertn., B.Mey. & Scherb. (Brassicaceae)	Meerrettich, Kren (S)	C	Roots		2 *	Tincture, honey, syrup, salve	Antibiotic, bronchitis, dry cough, bladder and kidney infections
*Arnica montana* L. (Asteraceae)	Arnica (T), Arnika, Bergwohlverleih (S)	W	Flowers, leaves, roots	2 *	9 *	Tincture, ointment oil infusion, salve	Rheumatism, musculoskeletal pain, muscle and bone injuries
*Artemisia absinthium* L. (Asteraceae)	Wermut, Bitterer Beifuß (S)	W	All parts of the plant		3 *	Tea, tincture	Digestive
*Artemisia umbelliformis* subsp. *umbelliformis* (Asteraceae)	Genepy, Genepi, Erba blanc (T)	W	Leaves, flowers	2		Schnaps	Digestive
*Artemisia vulgaris* L. (Asteraceae)	Beifuss, Besenkraut, Sonnenwendgürtel (S)	W	All parts of the plant		4 *	Tea, tincture, powder	Antiviral, antibacterial
*Betula pendula* Roth. (Betulaceae)	Betulla (T), Birke, Maibaum, Bedól (S)	W	Buds, sap, leaves, seeds	1 *	4 *	Birch sap, tea, tincture	Cholesterol, good for the kidneys, immune system booster, gives power, rheumatism
*Borago officinalis* L. (Boraginaceae)	Borretsch (S)	W, C	Flowers, leaves, seeds		1	Salad	Lifts the mood
*Calendula officinalis* L. (Asteraceae)	Ringelblume, Totenblume, Goldblume (S)	W, C	Flowers		10 *	Tea, salve, syrup, oil infusion	Wounds, scars, stomach and intestinal disorders, anti-inflammatory, skin-regenerating properties
*Carlina acaulis* L. (Asteraceae)	Silberdistel (S)	W	Roots		1 *	Tea, tincture, powder	Cold, fever, diuretic
*Centaurea benedicta* (L.) L. (Asteraceae)	Benediktenkraut (S)	W	Flowers, leaves		1 *	Tea, tincture	Stomach disorders
*Centaurea cyanus* L. (Asteraceae)	Fiordaliso (T), Kornblume (S)	W	Flowers	1	1	Tea, oil	Liver stimulator, good for digestion, skin disorders
*Cetraria islandica* (L.) Ach. (Parmeliaceae)	Lichen, Lichene islandico, Alga di montagna (T)	W	Aerial parts	7 *		Tincture, oil, ointment, boiled	Cough, bronchitis, COVID-19 prevention
*Corylus avellana* L. (Betulaceae)	Nocciolo (T)	W, C	Buds	1 *		Oil	Hemorrhoids
*Crataegus laevigata* (Poir.) DC. (Rosaceae)	Biancospino (T), Weißdorn, Mehlbeere (S)	W	Flowers, leaves, fruit, buds	1 *	1 *	Tea, tincture, powder, oil	Blood pressure balancer, calmant, diuretic, antispasmodic
*Crataegus monogyna* L. (Rosaceae)	Biancospino (T), Weißdorn, Mehlbeere (S)	W	Flowers, leaves, fruit, buds	1	1 *	Tea, tincture, powder, oil	Blood pressure balancer, calmant, diuretic, antispasmodic
*Dipsacus fullonum* L. (Caprifoliaceae)	Karde, Igelkopf, Weberdistel (S)	W	Roots		2	Tea, tincture, powder	Antibacterial, blood cleansing, detox
*Dryas octopetala* L. (Rosaceae)	Weiße Silberwurz, Stängellose Eberwurz (S)	W	Roots		1 *	Tea, tincture	Indigestion, appetite stimulator, laxative
*Elymus repens* (L.) Gould (Poaceae)	Quecke (S)	W	Roots		1 *	Tea	Anti-inflammatory, respiratory diseases
*Equisetum arvense* L. (Equisetaceae)	Equiseto, Erba cavallina, Coda cavallina (T), Ackerschachtelhalm, Zinnkraut (S)	W	Summer sprouts, leaves, stems	1 *	6 *	Tea, tincture, salve, powder, decoction	Osteoporosis, diuretic, kidney stones, vertebral and connective tissue strengthener
*Euphrasia officinalis* subsp. *pratensis Fr.* (Orobanchaceae)	Augentrost (S)	W	All parts of the herb		3 *	Tea	Eye disorders
*Ficus carica* L. (Moraceae)	Fico (T)	W, C	Buds	1		Oil	Digestion support supplement, stomachache
*Filipendula ulmaria* (L.) Maxim. (Rosaceae)	Spirea, Regina dei prati (T), Echtes Mädesüß, Geißbart (ST)	W	Aerial parts	1 *	3 *	Tea, powder, salve	As aspirin, cellulitis, cramps, diarrhea
*Gentiana lutea* L. (Gentianaceae)	Genziana, Enzian, Radis de genziana (T), Enzian (ST)	W	Roots, flowers	8	1 *	Tea, tincture	Depurative, stomach and intestinal disorders
*Geranium robertianum* L. (Geraniaceae)	Storchschnabel, Ruprechtskraut (S)	W	All parts of the herb		6 *	Tea, tincture, salve	Infertility, hormonal balance support supplement, heavy metal removal from the body
*Hedera helix* L. (Araliaceae)	Efeu (S)	W	Leaves		1	Tea, tincture	Cough
*Humulus lupulus* L. (Cannabaceae)	Ligaboschi, Luppol, Luppolo (T)	W	Fruit	8 *		External application	Sedative
*Hypericum perforatum* L. (Hypericaceae)	Iperico perforato, Erba di San Giovanni, Iperico (T), Johanniskraut, Blutkraut (ST)	W	Flowers	7 *	7 *	Tea, tincture, salve, oil	Skin burns, back pain, hemorrhoids, wounds, antidepressant, anti-wrinkle, antioxidant, anti-inflammatory, antiviral
*Juniperus communis* L. (Cupressaceae)	Wacholder, Kranewitt, Brusin (S)	W	Fruit, sprouts, roots,		1 *	Tea, tincture, syrup	Diuretic, kidney antiseptic, bladder inflammation, flatulence
*Lamium album* L. (Lamiaceae)	Weiße Taubnessel, Kuckucksnessel (S)	W	Flowers, leaves, roots		1 *	Tea, tincture	Menstrual cramps
*Lamium galeobdolon* (L.) L. (Lamiaceae)	Goldnessel, Gelbe Taubennessel (S)	W	Flowers, leaves, roots		1	Tea, tincture	Kidney and bladder infections
*Larix decidua* Mill. (Pinaceae)	Larice, Làres dalla resina (T)	W	Cones, buds, resin, flowers	11 *		Syrup, ointment, fomentation, topically applied	Cough, thorn removal, furuncles, burns
*Laurus nobilis* L. (Lauraceae)	Alloro (T)	C	Leaves, fruit	2 *		Oil	Dermatitis
*Lavandula angustifolia* Mill. (Lamiaceae)	Lavendel, Zöpfe (S)	W, C	Flowers		2 *	Tea, oil	Calmant, somniferum, headache, indigestion, acne, sunburn
*Leontopodium nivale* (Ten.) A.Huet ex Hand.-Mazz. (Asteraceae)	Edelweiß, Alpenedelweiß (S)	W	Flowers		2	Tincture	Good for memory, skin support supplement against premature ageing, sunscreen
*Leonurus cardiaca* L. (Lamiaceae)	Echtes Herzgespann, Bärenschweif (S)	W	Aerial parts		2	Tea, tincture, powder	Heart calmant
*Levisticum officinale* W.D.J.Koch (Apiaceae)	Liebstöckel, Maggikraut (S)	C	All parts of the plant		1 *	Powder, salt	Flatulence, digestive disorders
*Malva sylvestris* L. (Malvaceae)	Malva selvatica, Male va (T)	W	Flowers	10		Tea, topical application, bath, oil	Cystitis, constipation, skin rashes, vaginal lavages, anti-inflammatory, emollients, cough
*Matricaria chamomilla* L. (Asteraceae)	Camomilla (T), Kamille, Kummerblume, Mutterkraut (S)	W	Flowers	2 *	4 *	Oil infusion, salve, tea, ointment	Skin care, cramps, calmant, wounds, acne, bladder infections, stomach and intestinal disorders
*Melissa officinalis* L. (Lamiaceae)	Melisse, Bienenkraut (S)	C	Leaves		3 *	Tea, syrup, salve, oil	Nervous system calmant, heart support supplement; bladder disorders, herpes
*Mentha × piperita* L. (Lamiaceae)	Minze, Pfefferminze (S)	C	Leaves		2 *	Tea, syrup, oil	Headache, stomach and intestinal disorders
*Ocimum basilicum* L. (Lamiaceae)	Basilikum, Basilienkraut (S)	C	Leaves		1 *	Tea, tincture, pesto	Digestive disorders, flatulence
*Peucedanum ostruthium* (L.) W.D.J.Koch (Apiaceae)	Meisterwurz (S)	W	Roots		1 *	Tea	Gout, rheumatism, fever
*Phyllanthus niruri* L. (Phyllanthaceae)	Spacca pietra, Spacca muri (T)	C	Leaves, flowers	1		Decoction	Kidney stones
*Picea abies* (L.) H. Karst. (Pinaceae)	Aghi, Abete (T)	W	Leaves, buds	1 *		Fomentation, syrup	Respiratory system support supplement, cough
*Pimpinella major* (L.) Huds. (Apiaceae)	Bibernelle, Bockwurz (S)	W	Roots		1 *	Tincture, drying the root	Anti-inflammatory, antibiotic
*Pimpinella saxifraga* L. (Apiaceae)	Bibernelle, Bockwurz (S)	W	Roots		1 *	Tincture, drying the root	Anti-inflammatory, Antibiotic
*Pinus cembra* L. (Pinaceae)	Cirmolo, Cimbro (T)	W	Cones	3		Syrup	cough
*Pinus mugo* Turra (Pinaceae)	Pino mugo, Mughi (T)	W	Buds	8 *		Syrup, oil	cough
*Plantago lanceolata* L. (Plantaginaceae)	Piantaggine lanceolata (T), Spitzwegerich, Heilwegerich, Foie dei tai (S)	W	Leaves, roots, seeds	3	8 *	Tea, tincture, salve, bath additive, oil, topical application	Cough, insect bites, bronchial disorders, nervous system strengthener, eye support supplement, respiratory support supplement, hormone regulation
*Plantago major* L. (Plantaginaceae)	Breitwegerich, Wegtritt (S)	W	Leaves		2 *	Tea, salve	Cough, earache, sore throat foot blisters
*Polypodium vulgare* L. (Polypodiaceae)	Tüpfelfarn (S)	W	Roots		1 *	Tea, tincture	Mild laxative, diuretic
*Potentilla erecta* (L.) Raeusch. (Rosaceae)	Blutwurz, Rotwurz (S)	W	Roots		3 *	Tea, tincture, powder, salve	Throat disorders, stomach disorders, anti-inflammatory, astringent, wounds
*Quercus petraea* (Matt.) Liebl. (Fagaceae)	Eiche, Mosteiche (S)	W	Bark, acorns		1 *	Tea, tincture	Diarrhea, strengthener,
*Quercus robur* L. (Fagaceae)	Eiche, Mosteiche (S)	W	Bark, acorns		1 *	Tea, tincture	Diarrhea, strengthener,
*Rhodiola rosea* L. (Crassulaceae)	Rodiola rosea (T)	W	Roots	1		Tincture	Tonic
*Ribes nigrum* L. (Grossulariaceae)	Schwarze Johannisbeere, Ribisel (S)	W, C	Fruit, buds, leaves		1	Tea, tincture	Adrenal gland stimulator, anti-inflammatory, diarrhea, stomach pain
*Rosa canina* L. (Rosaceae)	Rosa canina (T), Hagebutte, Heckenrose (S)	W	Flowers, leaves, fruit, buds	2 *	2 *	Tea, powder, jam, oil	Immune system booster, allergies, vitamin C source, chronic bladder diseases, bronchitis, rheumatic complaints relief, arthrosis
*Rumex acetosa* L. (Polygonaceae)	Sauerampfer, Erba brusca (S)	W	Leaves		1 *	Soup, salad	Digestion stimulator, iron source
*Salix* spp. (Salicaceae)	Salice (T), Silberweide, Palmkätzchen, Salgar (S)	W	Bark, leaves, branches	2	1	Tea, tincture, salve, tincture	As aspirin
*Salvia officinalis* L. (Lamiaceae)	Salbei, Königssalbei, Zahnblätter (S)	W, C	Leaves		5 *	Tea, oil infusion, salt, toothpaste	Lymphatic system cleanser, excessive sweating, anti-inflammatory properties
*Salvia rosmarinus* Spenn. (Lamiaceae)	Rosmarin, Antonkraut, Weihrauchkraut (S)	C	Flowers, leaves		4 *	Oil infusion, salve, tincture	Calmant, heart and circulation strengthener, digestive system strengthener, flatulence
*Sambucus nigra* L. (Viburnaceae)	Sambuco (T), Holunder, Hollerbusch (S)	W	Flowers, fruit, roots	8 *	5 *	Tea, tincture, salve, syrup, Hollermulla (jam), deep-fried flowers	Cold, cough, fever, antioxidant, antiviral, Immune system strengthener
*Solidago virgaurea* L. (Asteraceae)	Goldrute, Pferdskraut (S)	W	All parts of the flowering plant		3 *	Tea, tincture, salve	Kidney stones, bladder disorders, fungal infections
*Sorbus aucuparia* L. (Rosaceae)	Eberesche, Vogelbeere (S)	W	Fruit		1 *	Jam, liqueur, syrup, powder, tea	Diarrhea, wounds, vitamin C source
*Symphytum officinale* L. (Boraginaceae)	Consolida (T), Beinwell, Wundallheil (S)	W	Roots, leaves	2	6 *	Tea, tincture, salve, ointment, oil	Swelling relief, muscular pain, wounds
*Taraxacum* sect. *Taraxacum* F.H.Wigg. (Asteraceae)	Denti de can, Denti de leone, Zicoria, Cicoria (T), Löwenzahn, Pustblume (S)	W	Flowers, leaves, roots, buds	5 *	12 *	Tea, tincture, powder, oil	Metabolism activator, dissolves deposits in the joints, cholesterol, secretory gland activator, depurative, anemia, diuretic, skin rashes
*Thymus serpyllum* L. (Lamiaceae)	Timo selvatico (T)	W	Leaves	6 *		Oil	Cough
*Thymus vulgaris* L. (Lamiaceae)	Thymian, Marienbettstroh (S)	W, C	Flowers, leaves, roots		8 *	Tea, tincture, salt, oil infusion	Bronchitis and dry cough, metabolism stimulator, menstrual cramp relief
*Tilia platyphyllos* Scop. (Malvaceae)	Tiglio (T), Linde, Steinlinde, Tiar (S)	W	Flowers, leaves, bark, buds	2	4 *	Tea, oil infusion	Fever, sore throat, diaphoretic properties, antistress, relaxant
*Tilia cordata* Mill. (Malvaceae)	Tiglio (T), Linde, Steinlinde, Tiar (S)	W	Flowers, leaves, bark, buds	2	4 *	Tea, oil infusion	Fever, sore throat, diaphoretic properties, antistress; relaxant
*Trifolium pratense* L. (Fabaceae)	Wiesenklee, Futterklee, Rotklee (S)	W	Aerial parts		1	Tea, tincture, salve, syrup	Eye infections, blood cleansing, mind calmant
*Tropaeolum minus* L. (Tropaeolaceae)	Kapuzinerkresse (S)	C	All parts of the herb		1	Decoration, salad	Good for the kidneys, support supplement for the immune system, gives power, rheumatism
*Tussilago farfara* L. (Asteraceae)	Huflattich, Bachblümlein, Fohlenfuß (S)	W	Flowers, leaves		2 *	Tea, tincture, fresh leaves	Mucolytic, cough, skin rashes and burns
*Urtica dioica* L. (Urticaceae)	Ortica (T), Brennnessel, Donnernessel, Piola (S)	W	Aerial parts, roots, seeds	6 *	12 *	Aerial part: tea, fresh juice, to make Knödel, Spätzle; Seed: to eat on bread or in salad; Root: to make a powder	Depurative, anemia, diuretic, anti-inflammatory, hair loss
*Valeriana officinalis* L. (Caprifoliaceae)	Baldrian (S)	W	Roots		1 *	Tea, tincture, bath additive	Sleep disorders, calmant
*Verbascum densiflorum* Bertol. (Scrophulariaceae)	Könikskerze, Fackelblume (S)	W	Flowers, leaves		3 *	Tea	Painful cough, mucolytic
*Verbascum thapsus* L. (Scrophulariaceae)	Tasso barbasso, Tasso verbasco (T)	W	Flowers	1 *		N.D.	Cold
*Veronica officinalis* L. (Plantaginaceae)	Echter Ehrenpreis (S)	W	All parts of the flowering plant		1 *	Tea, tincture	Digestive disorders, respiratory diseases, rheumatism
*Viola odorata* L. (Violaceae)	Veilchen, Wohlriechendes (S)	W	Flowers		1 *	Tea, tincture, syrup, vinegar	Bronchitis
*Viola tricolor* L. (Violaceae)	Stiefmütterchen (S)	W	All parts of the flowering plant		1 *	Tea, tincture	Cough, acne

**Table 2:** Main characteristics of our sample and the study areas.

Groups	Val di Sole	Überetsch–Unterland
Ecological environment of the area	Forested area with mainly spruce (*Picea abies* (L.) H.Karst.), fir (*Abies alba* Mill.) and stone pine (*Pinus cembra* L.) at higher altitudes; birch (*Betula* spp.), elder (*Alnus* spp.) and mixed shrubs at lower altitudes near pastures; large mountain pastures and cultivated areas, with mainly fruit trees	Area equally distributed between forests and agricultural surfaces (fruticulture and viticulture)
Altitudinal range (Highest village and altitude)	700–3757 m a.s.l. (Peio, 1200 m a.s.l.)	217–2439 m a.s.l. (Aldino, 1225 m a.s.l.)
Climate type (Koppen-Geiger)	Warm-summer humid continental climate	
Number of inhabitants of the studied area	Approximately 15,000	Approximately 76,000
Main economic sectors	Tourism, followed by animal husbandry and, in the lower valley, fruticulture	Mainly viticulture, fruit cultivation (mostly apple production), and tourism
Language and dialect	Italian, Solandro (local dialect)	German, South Tyrolean (local dialect), Italian
Historical background	Until 1815: Prince-Bishopric of Trent 1815–1918: part of the Habsburg Empire 1919: Paris Peace Conference annexed Trentino and South Tyrol to the Kingdom of Italy	Until 1919: Habsburg Empire 1919: Paris Peace Conference annexed Trentino and South Tyrol to the Kingdom of Italy 1946: Being mainly a German-speaking area, it was moved from Trentino to South Tyrol

**Table 3:** Main characteristics of the four studies including the original data we collected and the published data for comparison [* SSI = semi-structured interviews].

	Trentino		South Tyrol	
Data	Cappelletti and Fanzago, 1989 [38]	Our data (2022)	Pick-Herk, 1995 [39]	Our data (2022)
Collection period	1978–1979	2022	Presumably, 1994	2022
Method	SSI *	SSI *	SSI *	SSI *
Number of interviews	N.D.	22 (average age 59 years old)	83	30 (average age 48 years old)
Interviewees	Local elderly individuals (only knowledge orally obtained was documented)	Local people	Local plant experts, including midwives, monks, and farmers	Local people
Included wild and cultivated plants	yes	only wild	yes	yes

## Data Availability

All data are provided in the main article text.
